# Poisons

**Published:** 1874-06

**Authors:** 


### POISONS.

Poisons either burn or give other discomfort in passing down the throat; these are organic poisons, and are metallic, destroying the delicate lining of the parts along which they pass and causing inflammation more or less painful and dangerous, including all strong acids. There are other poisons which may be swallowed unawares as laudanum, paregoric, morphine and the like.

As the time for saving life in case of poisoning sometimes passes within five minutes or less, every head of a family and every intelligent person should have some general principles of action, with coolness of manner, so as to give the power to act advantageously in any emergency.

If an alkaloid—as opium, or morphia, or other anodyne—has been taken, all of which produce dulness of every grade to stupor and insensibility, the first point is to get it out of the stomach as instantaneously as possible; put a heaping teaspoonful of salt and as much ground mustard in half a glass of water, stir it and drink instantly. In a moment of time violent vomiting takes place, which should be promoted afterwards by drinking warm water, which further dilutes the poison. In a few moments drink freely of strong coffee, this helps to antagonize any of the poison which may remain. If the patient seems dull or sleepy, he should be taken under the arms by two persons and made to walk actively until he is fully waked up; if that cannot be done, put him under the pump and let the water fall on his head, or pour it on his head while sitting up, from a distance of two feet, from a pitcher or bucket until he is fully awake; for if you do not wake him he dies. In case of organic or metallic poisoning, scalding the throat in its passage, the first great point is dilution; the most accessible thing is warm water, but warm milk is better, and liquid oils or hog's lard are better still, for they not only dilute but they soothe and shield the parts in a very grateful manner; keep on drinking them until free vomiting is induced; then swallow the whites of several fresh eggs, which combine with the most acrid poisons and form chemical compounds, harmless and inert, of any remnant of poison which may be left after the vomiting. But while these things are doing, send for a physician, and when he comes tell him all that has been done and show him what has been vomited.

For many poisons there are more specific antidotes than the above; but most of them are seldom at hand. The points instantly to be arrived at are, in painless poisons to get them out of the stomach instantly. If painful, dilute first, cover the burned parts with some soothing material as oils, and then get them out of the stomach. Strong coffee and whites of eggs are good for both kinds of poisons.

